# Origin of the biphase nature and surface roughness of biogenic calcite secreted by the giant barnacle *Austromegabalanus psittacus*

**DOI:** 10.1038/s41598-020-73804-8

**Published:** 2020-10-08

**Authors:** Antonio G. Checa, Elena Macías-Sánchez, Alejandro B. Rodríguez-Navarro, Antonio Sánchez-Navas, Nelson A. Lagos

**Affiliations:** 1 Departamento de Estratigrafía y Paleontología, Universidad de Granada, 18071 Granada, Spain; 2 Instituto Andaluz de Ciencias de la Tierra, CSIC-Universidad de Granada, 18100 Armilla, Spain; 3 Radboud University Medical Center, Radboud Institute for Molecular Life Sciences, 6500 HB Nijmegen, The Netherlands; 4 Max Planck Institute of Colloids and Interfaces, 14476 Potsdam, Germany; 5 Departamento de Mineralogía y Petrología, Universidad de Granada, 18071 Granada, Spain; 6 Centro de Investigación e Innovación para el Cambio Climático, Facultad de Ciencias, Universidad Santo Tomás, Santiago, Chile

**Keywords:** Mineralogy, Biomaterials, Structural materials, Nanoscale biophysics

## Abstract

The calcite grains forming the wall plates of the giant barnacle *Austramegabalanus psittacus* have a distinctive surface roughness made of variously sized crystalline nanoprotrusions covered by extremely thin amorphous pellicles. This biphase (crystalline-amorphous) structure also penetrates through the crystal’s interiors, forming a web-like structure. Nanoprotrusions very frequently elongate following directions related to the crystallographic structure of calcite, in particular, the <− 441> directions, which are the strongest periodic bond chains (PBCs) in calcite. We propose that the formation of elongated nanoprotrusions happens during the crystallization of calcite from a precursor amorphous calcium carbonate (ACC). This is because biomolecules integrated within the ACC are expelled from such PBCs due to the force of crystallization, with the consequent formation of uninterrupted crystalline nanorods. Expelled biomolecules accumulate in adjacent regions, thereby stabilizing small pellicle-like volumes of ACC. With growth, such pellicles become occluded within the crystal. In summary, the surface roughness of the biomineral surface reflects the complex shape of the crystallization front, and the biphase structure provides evidence for crystallization from an amorphous precursor. The surface roughness is generally explained as resulting from the attachment of ACC particles to the crystal surface, which later crystallised in concordance with the crystal lattice. If this was the case, the nanoprotrusions do not reflect the size and shape of any precursor particle. Accordingly, the particle attachment model for biomineral formation should seek new evidence.

A widespread feature of biominerals is the marked roughness of their surfaces, which consist of amalgamated nanoprotrusions of varying sizes, always less than a few hundred nm. This nanostructure was first recognised with scanning electron microscopy (SEM) in the platelets of nacre^1,2^, although it was interpreted as fibrillar. Later, atomic force microscopy (AFM)^3^ revealed that the (etched) surfaces of septal nacre and mineral fibres of *Nautilus* and *Spirula* respectively consist of tightly packed nanoprotrusions (originally termed ‘granules’), measuring tens to hundreds of nm in diameter. This nanostructure was confirmed with AFM to be characteristic not only of nacre of bivalves and gastropods^4–7^, but also of other calcitic and aragonitic materials secreted by mollusks^8^, other invertebrates (sponges^9^, corals^10^, echinoderms^11^) and even vertebrates (fish otoliths^12^, avian eggshells^13^). Hitherto, the only biominerals not displaying that kind of surface roughness are coccoliths^14,15^. The same was invoked for the vaterite spicules of the ascidian *Herdmania momus*^16^, although they were later shown to have a nanoparticulate structure^17^. The dorsal arm plates of the brittlestar *Ophiocoma wendtii* contain calcite lenses most likely lacking surface roughness^18^. Accordingly, the rough surface quality is widely used as a diagnostic feature for biominerals^19,20^.

Besides the rough appearance, different authors (beginning with Dauphin^3^) have recognised the presence of two phases with different contrast in AFM tapping mode (phase imaging). There is a low contrast phase forming the bulk of the surface, and another high contrast (much darker) phase that extends as thin pellicles around and in between the nanoprotrusions. This is appreciated both at the surface and in the interior (e.g.^3,7^). While the first phase is clearly the crystalline phase, the high contrast phase has been hypothesised by some authors to be organic (e.g.^3,5–7,21,22^), and/or amorphous calcium carbonate (ACC)^8^ based only on AFM evidence; hence the term biphase applied here. Based on previous transmission electron microscopy (TEM) data on nacre^23^, it was proposed that similar pellicles in the sea urchin spines might consist of ACC^11^. Energy loss spectroscopy (EELS) coupled with TEM provided strong evidence that the amorphous phase in nacre consisted of ACC enriched in organic molecules^24^.

In summary, most biominerals have a nanostructure consisting of a rough outer surface with two unevenly distributed phases. This nanostructure is also found within the interior of the crystals and provides them with a ‘frothy’ appearance in polished section (e.g.^7^). Nevertheless, the amorphous pellicles are not continuous all around the crystalline nanoprotrusions, which, in this way, are continuous with each other^8,22^.

Since Towe and Lowenstam^25^, there has been increasing evidence indicating that biominerals in metazoans are formed from the corresponding amorphous precursor (see reviews in^26–28^). This is particularly well documented in the calcium carbonate biominerals (e.g. ^21,29–37^), which are by far the most common. Moreover, when synthesising calcite in vitro from ACC, it was observed that the crystals exhibited the surface roughness (usually referred to as ‘nanoparticulate’ structure) typical of biogenic crystals^38^. Based on this evidence, these authors interpreted this nanostructure as the result of a process of aggregation of ACC nanoparticles that subsequently crystallised into calcite. This process was termed crystallisation by particle accretion, CPA^39^. From then on, this view has been adopted as a tenet in biomineralization^19,20,40^.

Later on, TEM revealed that the front separating the amorphous and crystalline components in immature nacre (with a high content of amorphous component), takes a complex digitiform shape, indistinguishable from the surface roughness commonly observed in biominerals^24^. This led to the proposal that this nanostructure results from the progressive transformation of ACC into aragonite. These results, together with those of Checa et al.^22^, who found that similar nanoreliefs in nacre elongate and/or align in parallel to the *a*-axis of aragonite, strongly indicate that these features have clear crystallographic control, and do not represent aggregation units.

Acorn barnacles (order Sessilia, class Cirripedia) are sessile crustaceans that encase their soft body in a series of lateral plates, which are extensively calcified. This is unlike malacostracan crustaceans, in which the organic fraction predominates over the mineral one. Another important difference is that in malacostracans the mineral component is mainly amorphous calcium carbonate (ACC) and only a minor fraction of calcite^41,42^, whereas in cirripeds calcification is by means of calcite. There is, in addition, a minority organic phase composed of α-chitin and proteins^43–45^. The microstructures of a wide variety of cirripeds were studied by Bourget^46,47^. He described the granular microstructure composing the interior of the plate paries and basal plate, and the fibrillar crystals forming the radius margin. Similar granular units were also described in the giant barnacle *Austromegabalanus psittacus*^48^ in both the outer and inner surfaces of the parietes and basal plate while the sheath consisted of subparallel oriented lamellae, made of small equiaxial grains. Grains and rhombohedra were also observed in the base plate of *Balanus albicostatus*^49^ and the scutum and the wall plates of *B. amphitrite*^50^. Recently, the microstructure of two balanid species (*A. psittacus*, *Perforatus perforatus*) was described in detail^45^. The bulk of the plates is made of calcite grains, ranging in shape from anhedral to euhedral (rhombohedral), and in size from 0.5 to 10 µm. There is a minority fibrous microstructure found only at the growth fronts of the crenulations of the alae and radii. The crystallography of the wall plates of balanid cirripeds was studied by X-ray diffraction (XRD)^48^ and electron backscatter diffraction (EBSD)^49,51,52^. The latter study^52^ showed that the calcitic granular material composing the lateral plates of *A. psittacus* is much more ordered crystallographically than it looks at first glance. Grains grow epitaxially onto each other, thus producing crystallographically coherent columnar regions oriented in the local thickening direction of the plates. Crystallographic ordering is obtained by competition between neighbouring columnar regions. In this study, we will focus on individual grains. Our observations on *A. psittacus* and other species (*P. perforatus*, *Austrominius modestus*) reveal, for the first time, that grains have a conspicuous surface roughness, which is more marked than in other groups (e.g. molluscs). In addition, the protrusions frequently elongate along consistent orientations, something never observed previously in biocalcites. This latter feature is striking since the CPA hypothesis does not advocate any defined distribution or shape. Accordingly, we have studied the morphology, composition, crystallography and growth pattern of these grains, as well as the distribution, morphology, and crystallographic orientations of their accompanying nanoprotrusions using various techniques (SEM, TEM, EBSD, AFM, XRD…) in the balanid *A. psittacus*. The final goal is to establish a model regarding the origin of the nanoprotrusions. Our conclusions have profound implications as to how crystallization of ACC into calcite happens in biogenic calcite.

## Results

### SEM

**Figure 1:**
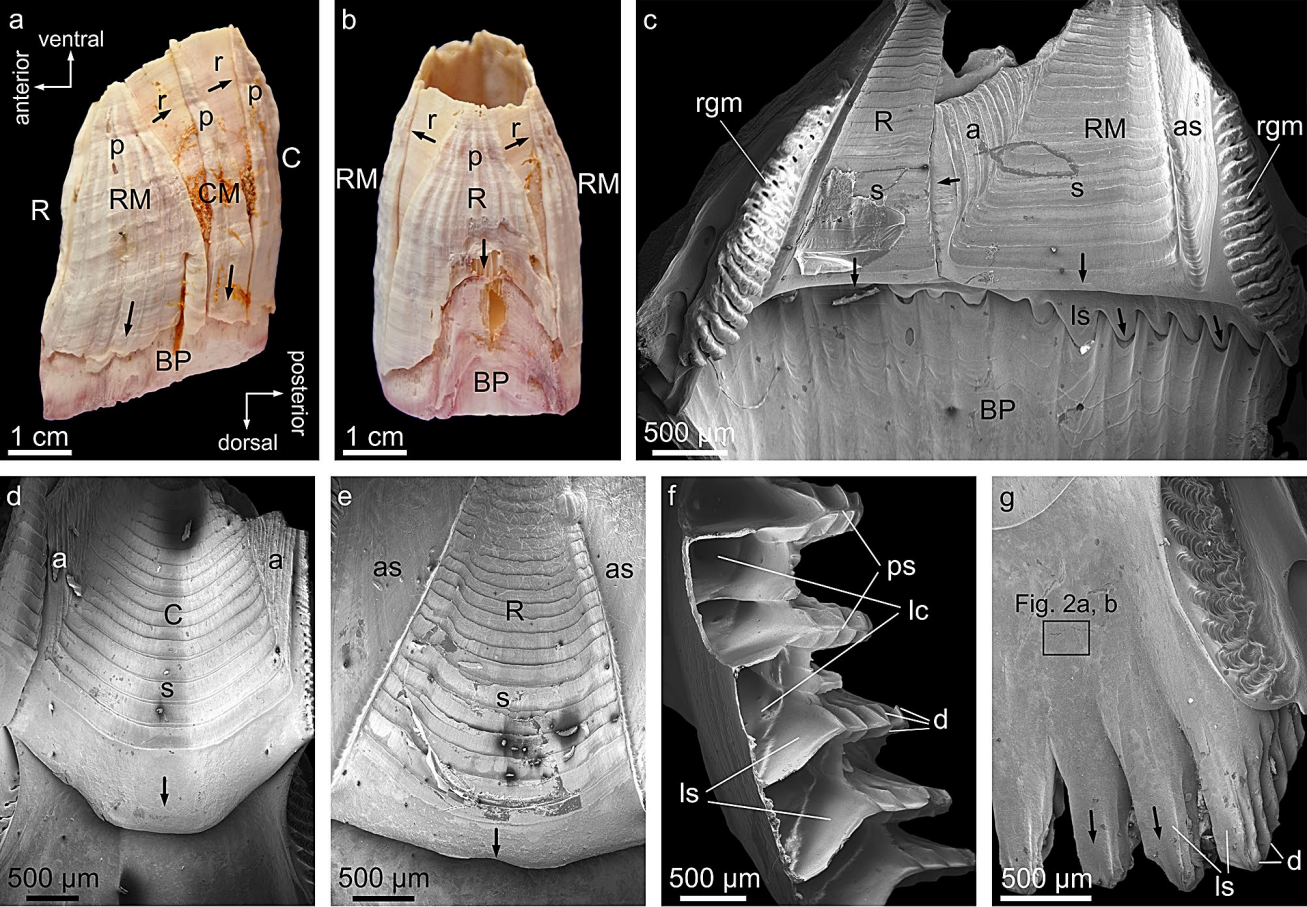
Main morphological elements of the exoskeleton of *A. psittacus*. (**a**, **b**) Left and anterior external views of a complete specimen. The anatomical directions are indicated. (**c**) Internal view of the rostral and rostromarginal plates. (**d**) Internal view of the carina. (**e**) Internal view of the rostrum. (**f**) Dorsal growth margin of the paries. (**g**) Internal view of the dorsal margin of a rostromarginal plate. The framed area is similar to the one from which the images of Fig. 2a and b were taken. Arrows indicate the growth directions of particular elements. *a* ala, *as* articular surface, *BP* base plate, *C* carina, *CM* carinomarginal plate, *p* paries, *ps* primary septum, *R* rostrum, *rgm* growth margin of the radius, *RM* rostromarginal plate, *s* sheath. The figure was generated with the software CorelDRAW Home & Student X8 (https://www.coreldraw.com/la/product/home-student/) by AGC.

The shell of *A. psittacus* (Balanidae, Cirripedia) has bilateral symmetry and consists of six calcitic plates, named, from anterior to posterior: rostrum, two symmetrical rostromarginals, two carinomarginals and carina (Fig. 1a,b). Externally, the plates consist of two structures: a paries, which has a corrugated surface and which grows towards the substrate, and one (rostromarginal, carinomarginal; Fig. 1a) or two (rostrum; Fig. 1b) radii, with smooth surfaces, which grow toward the carina. The growth margins of the radii develop horizontal crenulations with dendritic margins (Fig. 1c). The carina has no radii. In addition, there is a basal plate, as in all Balanidae^53^, for adhesion to the substrate (Fig. 1a–c). Internally, the plates develop a distinctive sheath (Fig. 1c–e), which extends from the venter toward the dorsum to a variable extent. The sheath of the carina extends laterally into two symmetrical alae (Fig. 1d), whereas those of the carinomarginal and rostromarginal plates only have one ala each (Fig. 1c). The rostrum has no alae (Fig. 1c,e). All alae grow toward the rostrum and they all fit into a smooth area adjacent to the margin of the sheath of the opposing plate (the articular surface for the adjacent ala) (Fig. 1c,e). Other distinctive elements are the longitudinal septa, protruding from the dorsal margin of the paries (Fig. 1c,f,g), which interlock with the radially-arranged primary tubes of the base plate, one-to-one (Fig. 1c). They have branching growth margins consisting of radial primary septa and diverging denticles (Fig. 1f). The longitudinal septa separate the longitudinal canals which run in a dorsoventral direction within the paries interior. Further details on the distributions and morphologies of plates can be found in^45,54,55^.

**Figure 2:**
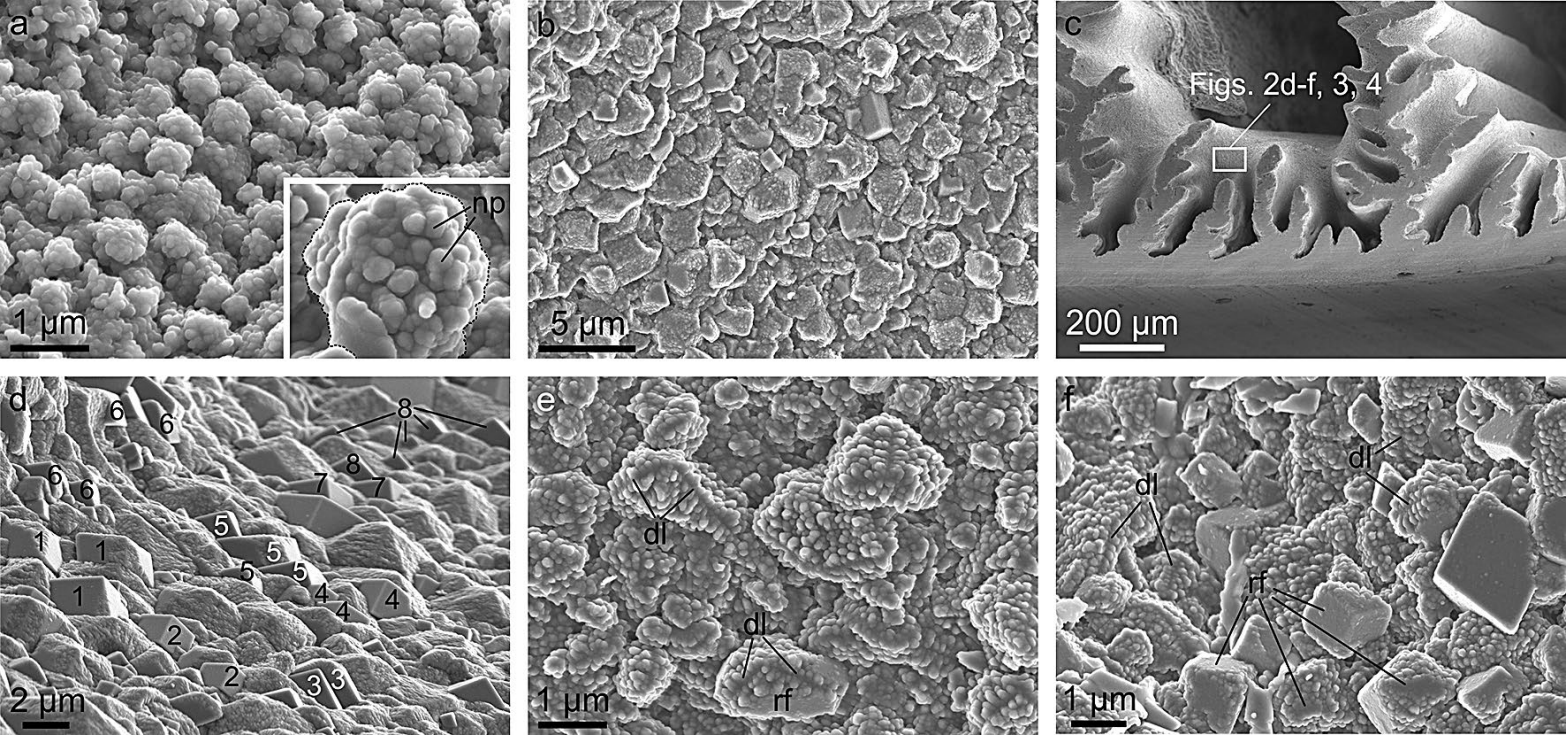
SEM views of the calcitic grains making up the plates. (**a**) Internal view of the dorsal half of a rostromarginal plate (a similar area is indicated in Fig. 1g), showing rounded, small-sized grains. The inset shows a grain (delineated with broken line) with the surface nanoprotrusions indicated. (**b**) View of the internal surface of a different plate. The surface is studded with grains, ranging from irregular to rhombohedral. (**c**) View of the growth margin of a rostrum, indicating an area similar to that from which the mentioned images were taken. (**d**) Growth surface of the margin of a radius showing variously shaped grains, from rounded to rhombohedral. Grains with similarly oriented rhombohedral surfaces are identified with the same numbers. (**e**, **f**) Views similar to (**d**) showing the great diversity in grain morphology, from irregular to neatly rhombohedral. Lines of divergence of nanoprotrusions are frequent. *dl* divergence lines, *rf* rhombohedron face. The figure was generated with the software CorelDRAW Home & Student X8 (https://www.coreldraw.com/la/product/home-student/) by AGC.

The bulk of the shell plates has a microstructure made up of calcite grains of varying sizes and shapes (Fig. 2), usually defined as granular. Only the growth margins of both the radii and alae consist of calcitic fibres oriented perpendicular to the growth margin^45,52^ (Supplementary Fig. S1). This study is focused on the granular microstructure. Average grain size changes according to the location on the plate surfaces. The grains studding the internal growth surfaces of the plates are noticeably small (< 1–4 µm) (Fig. 2a,b). The largest sizes are found at the troughs formed both at the growth ends of the longitudinal septa (Fig. 1f) and at the growth surfaces of the radii (Figs. 1c and 2c). Here, grains are > 2 µm and can reach up to 8 µm (Fig. 2d). In a particular area, the sizes of grains also change, sometimes drastically (e.g. Figure 2d).

The shapes of grains are extremely variable, from rounded or irregular to typically rhombohedral (Fig. 2a,b,d–f). Based on the shapes, the fully grown rhombohedra are of the most common {104} form (Fig. 2d,f). The observation of the growth surfaces shows how crystals grow onto and interpenetrate each other (Fig. 2d–f). In addition, clusters of nearby grains may have parallel rhombohedral faces, i. e. they are cooriented (Fig. 2d).

**Figure 3:**
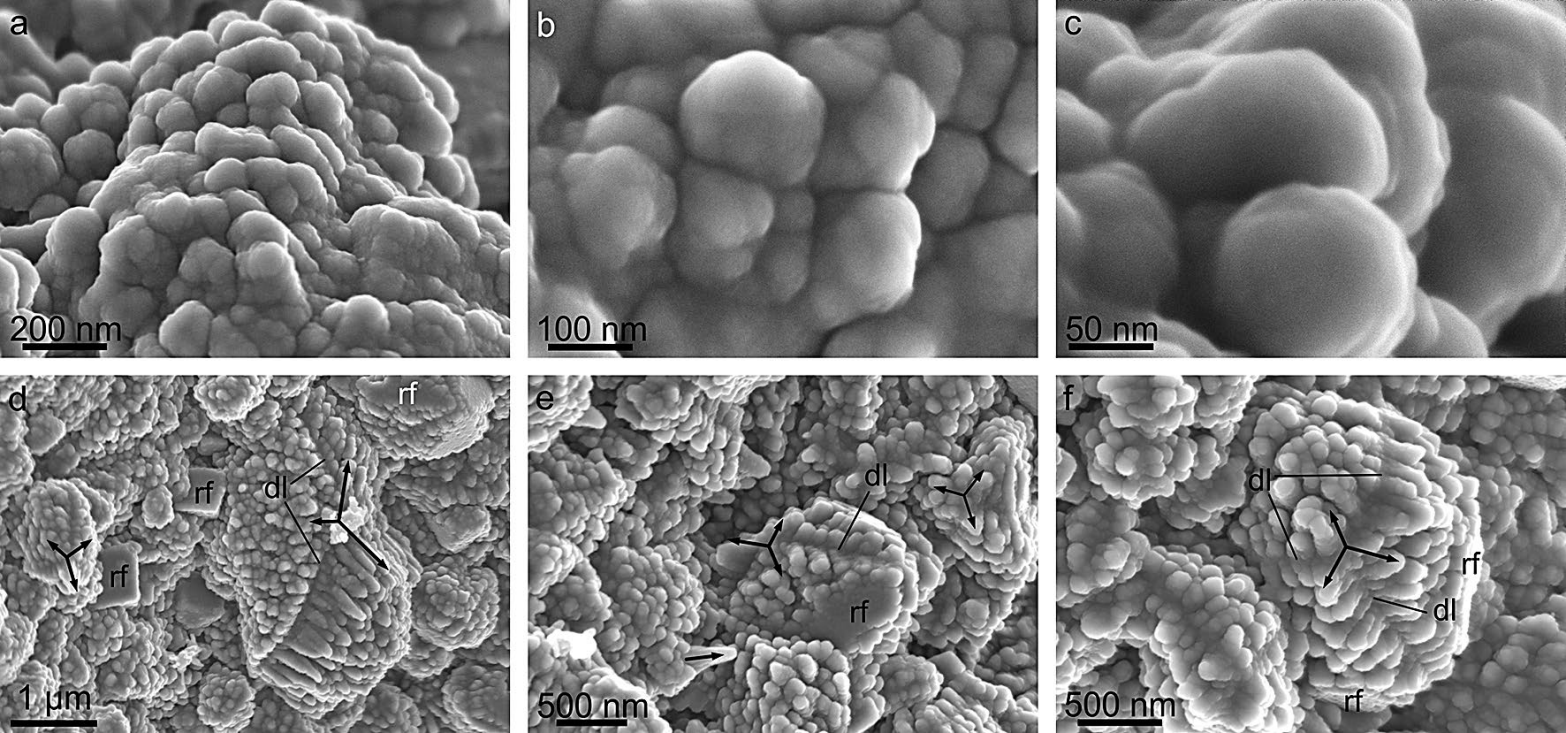
Morphology of nanoprotrusions present on the grain surfaces. (**a**–**c**) Progressive close-ups showing the complex morphologies of the surfaces. (**d**-**f**) Examples of elongated nanoprotrusions present in grains. Arrows indicate the approximate elongation axes of the nanoprotrusions. The nanoprotrusions corresponding to each growth sector of the grains have parallel elongation axes. These change at similar angular distances across the divergence lines. *dl* divergence line, *rf* rhombohedron face. The figure was generated with the software CorelDRAW Home & Student X8 (https://www.coreldraw.com/la/product/home-student/) by AGC.

**Figure 4:**
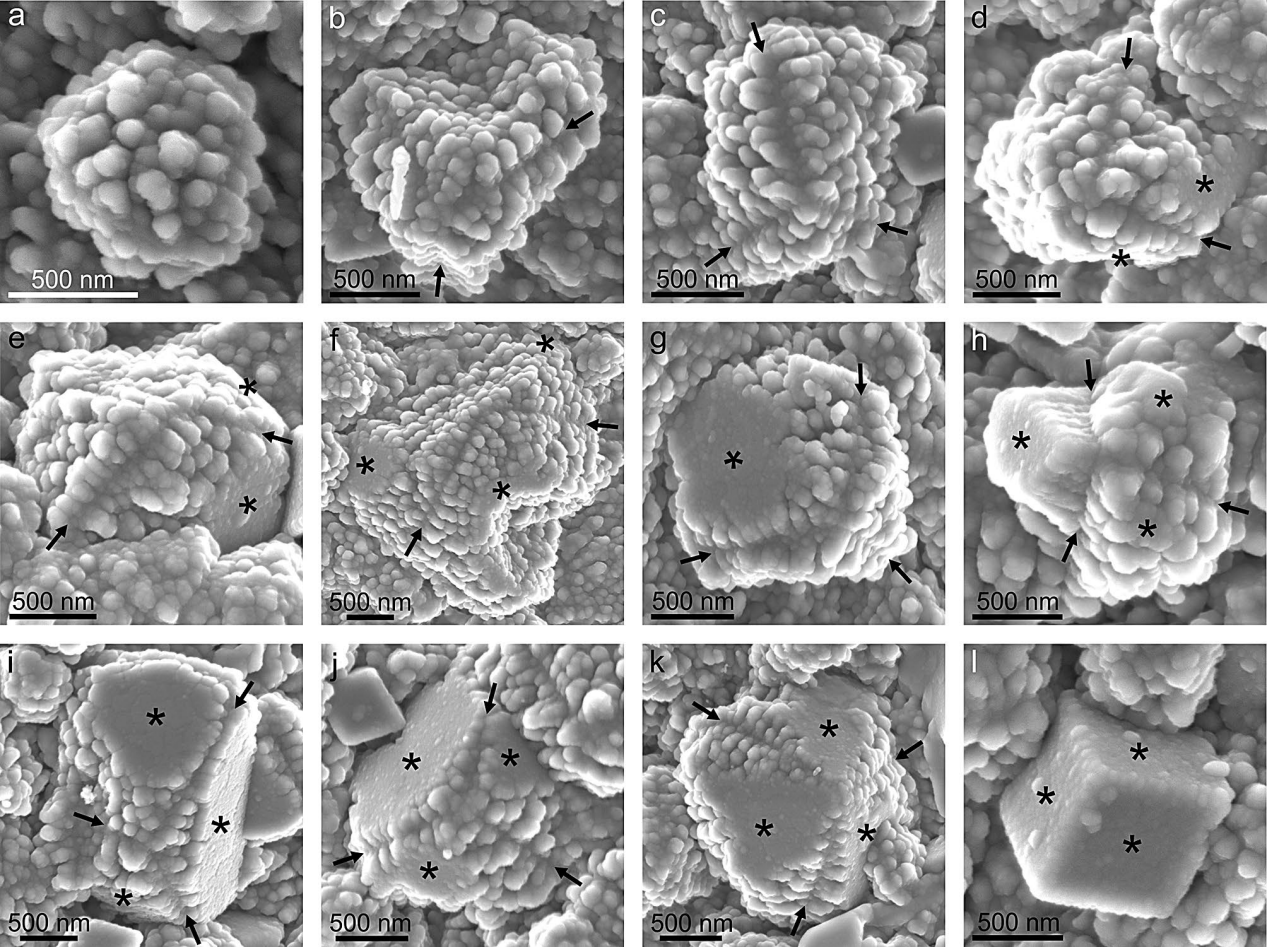
A complete morphological sequence of grains. (**a**) Irregular grain. (**b**, **c**) Irregular grains with lines of divergence. (**d**-**g**) Besides divergence lines, grains begin to develop rhombohedral faces. (**h**) Grain with three lines of divergence that meet at ~ 120°, and three incomplete rhombohedral faces (see indexation in Fig. 7). (**i**–**k**) Grains with neatly developed rhombohedral faces and incomplete edges. (**l**) Complete rhombohedron. Asterisks indicate rhombohedral faces and arrows point to lines of divergence of nanoprotrusions. The figure was generated with the software CorelDRAW Home & Student X8 (https://www.coreldraw.com/la/product/home-student/) by AGC.

Calcite grains display a very conspicuous surface roughness consisting of nanometer-sized protrusions (hence the term nanoprotrusion we apply herein) of variable sizes from ˂ 20 to > 150 nm, and ~ 100 nm on average (Fig. 2a, inset, e,f). This has also been observed in *Perforatus perforatus* (Balanidae, Balanoidea) and *Austrominius modestus* (which we reinterpret here as *Elminius kingii*) (Austrobalanidae, Tetraclitoidea) (Supplementary Fig. S2), and is possibly common in cirripeds. In detail, these nanoprotrusions are not discrete units and their surfaces are irregular, as if they in turn contained tinier nanoprotrusions, in a sort of nanoprotrusion-onto-nanoprotrusion or fractal-like surface pattern (Fig. 3a–c). There is a sharp contrast in surface roughness between the rhombohedral faces and the rest of the growth surfaces of the grains. The former faces are much smoother, and it is not possible to distinguish individual nanoprotrusions (Figs. 2f and 4). An important feature is that the nanoprotrusions of non-rhombohedral surfaces are frequently neatly elongated, sometimes in a dramatic fashion, forming rod- or matchstick-like structures (Figs. 3d–f, and 4b–k). Rod-like nanoprotrusions form parallel arrangements that are consistently at high angles to the rhombohedral faces. This is particularly evident in some grains with incomplete rhombohedral faces (Figs. 3e,f, and 4d,e,g,h,j,k). In top views of the forming rhombohedra, rod-like nanoprotrusions change their orientations by ~ 120° to accommodate to the changing orientation of the rhombohedral faces (Figs. 3d–f, and 4d–k). These changes in orientation take place along well-defined divergence lines, which, upon completion of the rhombohedron, correspond to its edges (Figs. 3d–f, and 4h–k).

It is possible to trace a sort of morphological sequence starting with irregular grains (Fig. 4a). Later, some grains develop lines of divergence of elongated nanoprotrusions (Fig. 4b,c), followed by incipient flat rhombohedral surfaces initiated at or close to their centres (Fig. 4d–f). Rhombohedral faces later extend toward the periphery (Fig. 4g–j). With edge formation, a rhombohedron is complete (compare Fig. 4j, and k to l). We assume that this is also the approximate transformation sequence of anhedral grains into rhombohedral. Every grain follows this sequence to a different extent and, judging from the observed crystalline habits, relatively few reach the end of this sequence.

### EBSD

This study only presents measurements in addition to those in our recent extensive EBSD study of the plates of *A. psittacus*^52^. In both horizontal (Supplementary Fig. S3a) and vertical sections (Supplementary Fig. S3b), the maps reveal the existence of evenly coloured (implying identical orientations) elongated areas with very irregular, jagged margins. These were named crystallographically coherent regions (CCRs)^52^. By comparing the shapes and extensions of CCRs (elongated, several tens of micrometres) and those of grains (equiangular, from less than one to a few micrometres), we conclude that the CCRs must be formed by many co-oriented calcite grains. CCRs orient their long axes more or less perpendicular to the growth surface and internal growth bands of the sheath (in black in Supplementary Fig. S3). By plotting the cell lattices onto the maps, it becomes clear that the *c*-axes are parallel to the elongation of CCRs and perpendicular to the growth surfaces. When growth bands suddenly change in curvature, CCRs diverge accordingly (Supplementary Fig. S3). 001 pole figures display good clustering with maxima oriented in the direction of growth of CCRs, whereas 100 maxima show arch-like distributions indicating no preferred orientation of the *a*-axes of calcite. EBSD results indicate that the grains (either anhedral, subhedral of euhedral) observed on the growth surfaces (Figs. 2a,b,d–f, 3d–f, and 4) have their *c*-axes more or less parallel to the viewing axis.

### AFM

**Figure 5:**
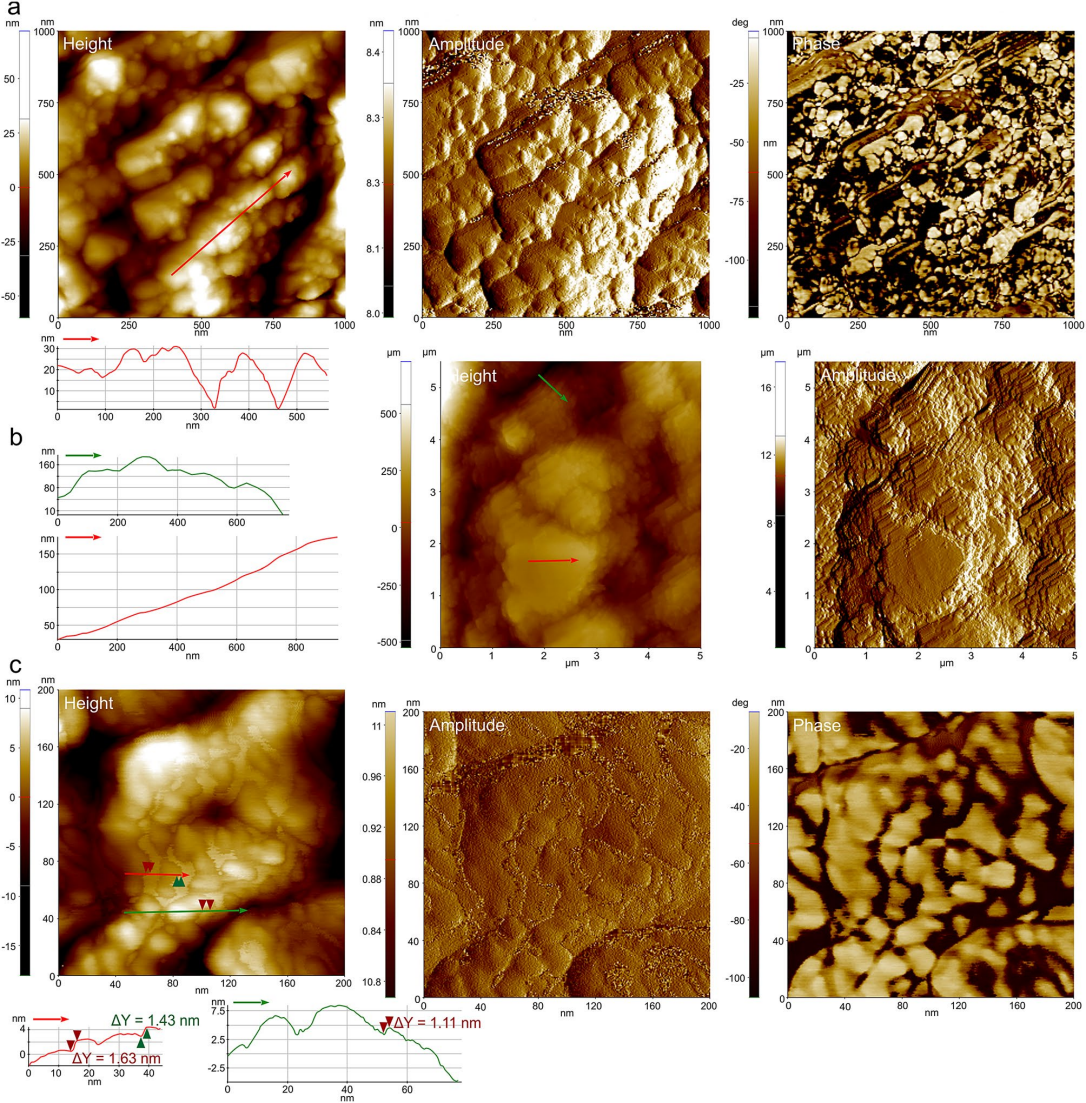
AFM analysis of calcitic grains. (**a**) View of a non-rhombohedral surface with elongated nanoprotrusions. The height and amplitude images, as well as the height profile, show minor nanoprotrusions superimposed onto the higher nanoprotrusions. The phase image shows the irregular distribution of the high-contrast phase. (**b**) View of a relatively large area, showing the difference in surface roughness between a rhombohedral (red profile) and a non-rhombohedral surface (green profile). (**c**) Detail of a non-rhombohedral surface showing the aspect of the amorphous pellicles and their irregular distribution. The height differences (ΔY) measured on the profiles correspond to the boundaries of the pellicles and indicate their approximate thicknesses. The panels were generated with the software Park Systems XEI 4.3 (https://park-systems-xei.software.informer.com/) and the figure was composed with CorelDRAW Home & Student X8 (https://www.coreldraw.com/la/product/home-student/) by AGC.

AFM images clearly show the surface roughness of the grains (Fig. 5a–c). Transects in height images show changes in the order of several tens of nm (20–40 nm) corresponding to individual nanoprotrusions, but with no defined amplitude (Fig. 5a–c). Height differences become reduced to a few nm (2–5 nm) on the much flatter rhombohedral faces (Fig. 5b, and Supplementary Fig. S4a,b). The elongation of nanoprotrusions can also be appreciated (Fig. 5a,b, and Supplementary Fig. S4c). There are two different materials in the phase images, one in a light colour (low contrast), and another, much darker in colour, which distributes as a kind of incomplete peel onto the low contrast phase, preferentially at depressions between nanoprotrusions (Fig. 5a,c, and Supplementary Fig. S4d). Transects reveal that height changes at the edges of these coats (i.e. their thicknesses) are in the order of >  > 1 nm to ~ 2 nm (Fig. 5c, and Supplementary Fig. S4a,c). The high contrast phase sometimes covers a relatively small proportion of the scanned surface, both in non-rhombohedral (Fig. 5c, and Supplementary Fig. S4d) and rhombohedral surfaces (Supplementary Fig. S4b). In other instances of both types of surfaces, this dark phase covers a much more significant proportion of the surface (Supplementary Fig. S4a,c). We have also observed the lines of divergence of nanoprotrusions described with SEM. They are typically straight and do not show any particular distribution of low versus high contrast phases (Supplementary Fig. S4d).

### TEM

**Figure 6:**
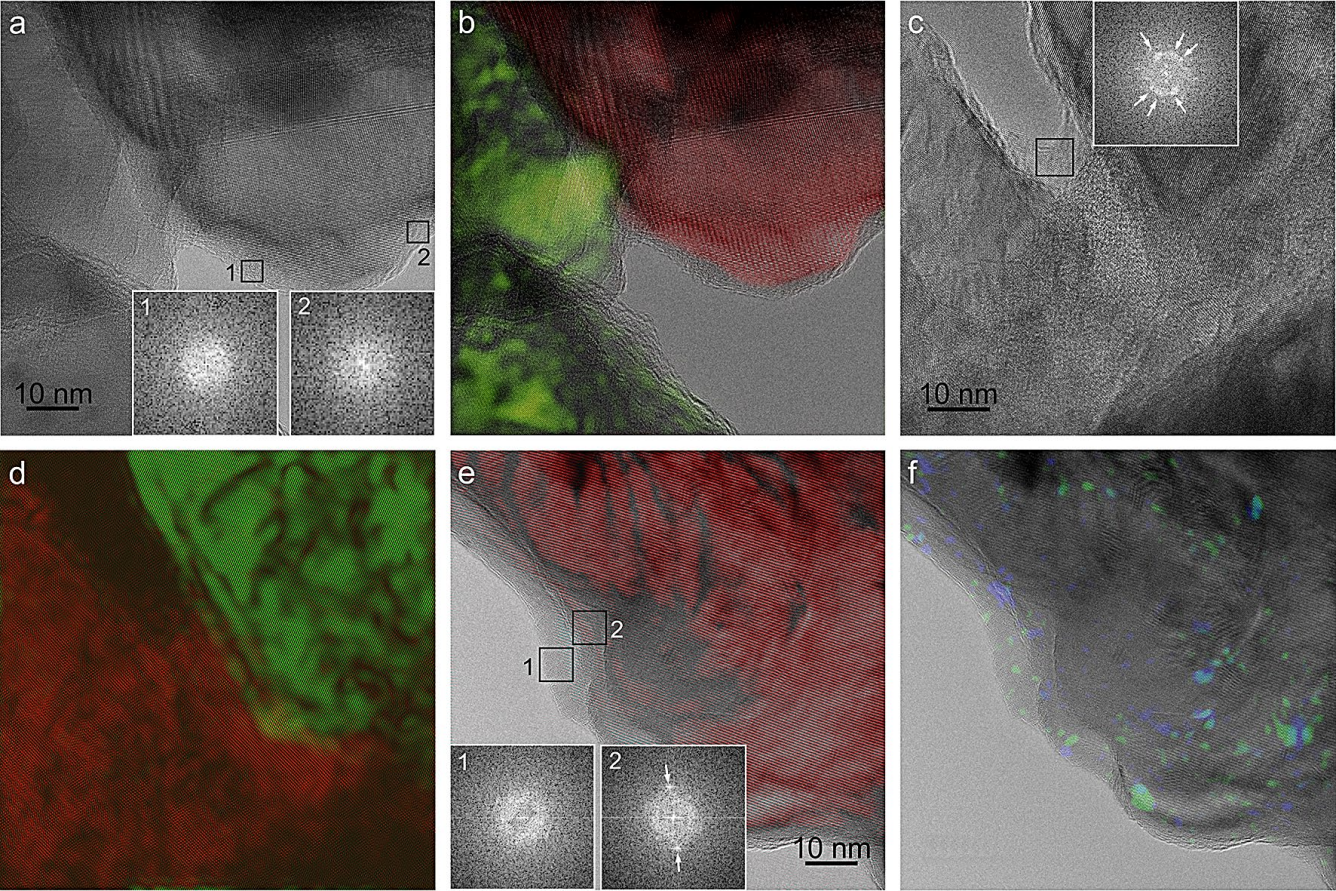
TEM of grains. TEM (**a**, **c**, **f**) and colour reconstructions of the crystalline areas (**b**, **d**, **e**), obtained by selecting the main reflections. Non-crystalline areas appear as thin marginal and internal stripes. FFTs 1 and 2 in (**a**) and 1 in (**e**) reveal the amorphous nature of the rims. Reflections (arrows) in FFT 2 in (**e**), taken in a more interior area, are indicative of crystalline material. The multiple reflections (arrows) in the FFT in (**c**) derive from recrystallization due to beam damage. The morphology of the boundaries between crystalline and amorphous domains is complex in all cases, particularly in the green domains of (**b**) and (**d**). The small colour areas in (**f**) correspond to recrystallizations induced by beam damage. Some of them occurred in the amorphous areas (compare (**e**) to (**f**)). The panels were generated with the software Gatan Digital Micrograph 3.4 ( https://www.gatan.com/products/tem-analysis/gatan-microscopy-suite-software) by EMS, and the figure was composed with CorelDRAW Home & Student X8 (https://www.coreldraw.com/la/product/home-student/) by AGC.

TEM imaging shows that most of the material is crystalline (Fig. 6a–f), but there are also non-crystalline areas, which are distributed as thin stripes both around the grain surfaces and within the interior (Fig. 6b,d,e). The thicknesses of the stripes change between 1 and 5 nm (rarely close to 10 nm, e.g. Figure 6e), and tend to be thicker at the grain margins. Despite the low resolution of fast Fourier transforms (FFTs) due to the reduced size of the areas, they demonstrate the amorphous nature of the rims (Fig. 6a,e). FFTs also reveal cases of recrystallization by beam damage (Fig. 6c). The stripes within the grain interiors are too thin to provide reliable FFTs. When the amount of non-crystalline material is particularly great (Fig. 6b, green domain), the complex aspect of the crystalline front is evident. By exposing the sample to beam radiation, it is possible to induce local recrystallization, both of the crystalline and the amorphous part (Fig. 6c,f), which implies that the latter contains ACC. Some of the non-crystalline areas may have been originally crystalline and became amorphised or recrystallised by beam damage. Since the colour reconstruction is based on the selection of particular reflections, some very minor recrystallised areas, whose reflections have not been selected, may still appear as non-crystalline (Fig. 6c,d). Nevertheless, most of the thin amorphous stripes revealed upon FFT analysis must have been original, as we always tried to minimise beam radiation. Moreover, beam damage, when induced, affects irregular areas (Fig. 6f), very different from the stripe-like non-crystalline areas observed here.

### Determination of ACC by X-ray diffraction (XRD) and thermogravimetric analysis (TGA)-differential scanning calorimetry (DSC)

Following the methodology described by Albéric et al*.*^56^, we combined XRD, TGA, and DSC to determine the wt% of ACC. XRD scan of the mixture of shell material and silicon (20%) as internal standard was used to determine the percentage of amorphous material using the Rietveld refinement method (Supplementary Fig. S5a). The estimated percentage of amorphous phases is 5.59 ± 0.8% over three replicates. The amount of water (0.57 ± 0.04%) and organic matter (1.23 ± 0.03%) over three replicates was determined from TGA curves (Supplementary Fig. S5b). By detracting the estimated ~ 1.8% of water + organic matter (with TGA) from the ~ 5.6% amorphous phase (XRD), the amount of ACC is *ca.* 3.8%. This amount has to be taken as approximate given the relatively high error associated to the techniques employed. A previous attempt with XRD and infrared spectroscopy did not reveal any significant amount of ACC in the balanid *Balanus amphitrite*^50^. Our calculation is well below previous estimations for other biogenic calcites: the sea urchin test plates and spines (~ 10% ACC) with similar methods^56^, sea urchin spines (~ 8 at.%) with ^11^C nuclear magnetic resonance (NMR)^11^, and bivalve calcite (~ 6.9 at.%) with X-ray absorption near edge structure (XANES)^57^. From DSC curves, heat flow peaks associated with organic matter combustion from 290 °C to 500 °C and carbonate thermal decomposition from 600 to 900 °C were detected (Supplementary Fig. S5b). However, no clear peak associated with ACC to calcite crystallization (expected at ~ 200 °C) was detected, possibly due to the low amount of ACC.

## Discussion

### The mineralization compartment

The massive granular microstructure of barnacles forms the bulk of the plates and is made of calcite grains which, on the growth surfaces, protrude above each other by several microns, making it possible to easily differentiate the shapes of particular grains (Figs. 2a,b,d–f, 3d–f and 4). This is unlike molluscs and brachiopods, in which irregularities on growth surfaces are well below the micron size. This is because the growth surfaces are levelled off by the mantle epithelium cells, which are at very small distances from the forming crystals^58,59^. In cirripeds, there must not be such extreme space restriction, and the distance between the forming crystals and the mantle must be of the order of several microns. Calcite grains can grow freely from a solution in this space. The same likely accounts for the particularly well developed nanoprotrusions in cirripeds as compared to the aforementioned groups, where nanoprotrusions are much less pronounced. Given the relatively small grain size observed (< 1–8 μm), either there must be a high nucleation flux (compatible with relatively high supersaturation), or specific organic components are promoting intense nucleation at lower supersaturation^60^. At present, it is not possible to decide between these two possibilities.

### Nature and distribution of the amorphous phase

The distinction between a crystalline and an amorphous phase at the nanoscale has long been known in both biocalcite and bioaragonite (e.g.^8^). This also applies to the mode of distribution of the amorphous phase as pellicles around the nanoprotrusions of the crystalline phase. AFM reveals that pellicles are discontinuous and very thin (<< 1–2 nm) and form a variable percentage of the whole surface (Fig. 5a,c, and Supplementary Fig. S4a,c,d). We do not know if the observed distribution is the original distribution or if, despite our specimens having been sampled alive, these pellicles were originally much more extensive and deteriorated during sample manipulation, due to their lability. The presence of surfaces with extensive dark covers (Supplementary Fig. S4a,c) weakly suggests that this could be the original condition. AFM observations on polished sections by other authors reveal that the pellicles are present within the material interior^3,7,61^. Our TEM observations match the AFM observations relatively well. We can easily correlate the high-contrast pellicles observed under AFM with the amorphous stripes observed under the TEM within the grain interiors. Since the external pellicles and the internal stripes look identical under the AFM (e.g.^3,7,61^) we infer that both share the same nature. Internal and external pellicles, and amorphous rims were previously observed with TEM on the surfaces of both biogenic calcite^21^ and aragonite^23^ grains. These pellicles were interpreted as organic by former authors (e.g.^3,5–7,21,22^). However, a Ca signal was found with EELS on similar pellicles in nacre, although lower than in the crystalline part^24^, which led these authors to infer that they must contain some ACC. Our recrystallization experiments, as well as those of^23^ on similar amorphous cortexes in nacre, point in the same direction. The sensitivity of our material has prevented us from performing similar EELS analyses. In summary, present data imply that the amorphous phase is composed of a mixture of ACC enriched in organic molecules. All this is consistent with the low, but significant 3wt% ACC estimated by XRD and TGA–DSC.

### Crystallography and growth of calcite grains and their nanoprotrusions

**Figure 7:**
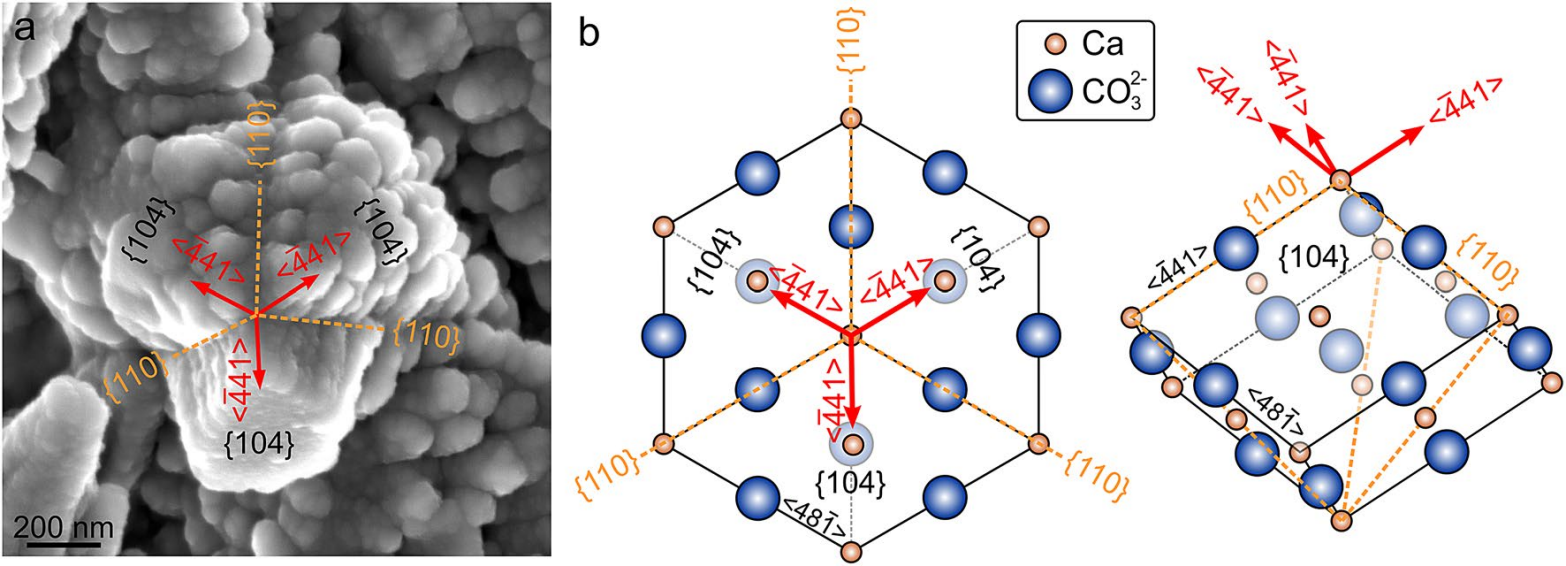
Indexation of the elongated nanoprotrusions by comparison with the calcite rhombohedron. (**a**) Incomplete rhombohedral grain showing elongated nanoprotrusions. (**b**) Theoretical calcite rhombohedron seen in plan and in lateral views. Both the grain in (**a**) and the plan view in (**b**) (left) have approximately the same orientation. The comparison allows us to index the main morphological elements present in (**a**). It is particularly significant that the nanoprotrusions elongate along the <− 441> directions. The figure was generated with the software CorelDRAW Home & Student X8 (https://www.coreldraw.com/la/product/home-student/) by AGC.

EBSD data show that the *c*-axes of grains are, in general, perpendicular to the growth surfaces. This means that when the growth surfaces are imaged in-plane (Figs. 2, 3 and 4), the *c*-axes of grains must be pointing towards the observer. In the case of rhombohedra (even if unfinished), the corner pointing toward the observer is where the *c*-axis pierces through the rhombohedron (Figs. 2e,f, 3d–f, and 4b–l). In those grains where {104} faces have not yet begun to form or are incomplete, the rod-like nanoprotrusions diverge from neat lines (Figs. 3d–f, and 4b–k) which, with growth, become rhombohedron edges (Fig. 4l). Accordingly, these lines are intersections of the {110} planes with the grain (Fig. 7a). When the nanoprotrusions are notably elongated, they do so at high angles to the {104} faces, at the same time that they acquire a slight ascending trajectory (Figs. 3e,f, 4d–h,j, and 7a). If we compare such grains with the theoretical calcite rhombohedron (compare Fig. 7a to b), it becomes clear that the axes of elongation of nanoprotrusions coincide with the rhombohedron edges, that is, the <− 441 > directions of calcite.

Formerly, we established a theoretical growth sequence starting from fully irregular grains followed by grains with neat lines of divergence of nanoprotrusions (Fig. 4a–c). Later, some of the elongated nanoprotrusions stop growing and their growth ends become flat and level off, forming the centres of {104} rhombohedral faces (Fig. 4d–f). With further growth, the peripheral addition of nanoprotrusions contributes to the extension of rhombohedral faces towards the edges, which are the last elements to be finalised (Fig. 2g–l). This growth mode is quite unlike that of inorganic calcite, in which crystals grow by the addition of new molecular planes by island growth or screw dislocation (e.g.^62,63^).

### Model for the development of the biphase nature and surface roughness

As stated above, nanoprotrusions grow preferentially along the <− 441 > directions of calcite. These directions are particularly significant because they are the strongest periodic bond chains (PBCs) in calcite (e.g.^64,65^), i.e. uninterrupted chains of strong bonds formed in the crystal lattice. Interestingly, a similar conclusion was reached in biogenic aragonite, where similar nanoprotrusions align and grow along the <100> direction^22^, which is the strongest PBC in aragonite. Accordingly, nanoprotrusion development is crystallographically-controlled, and any explanation as to their origin should contemplate the anisotropy of the crystallization forces during crystal growth.

**Figure 8:**
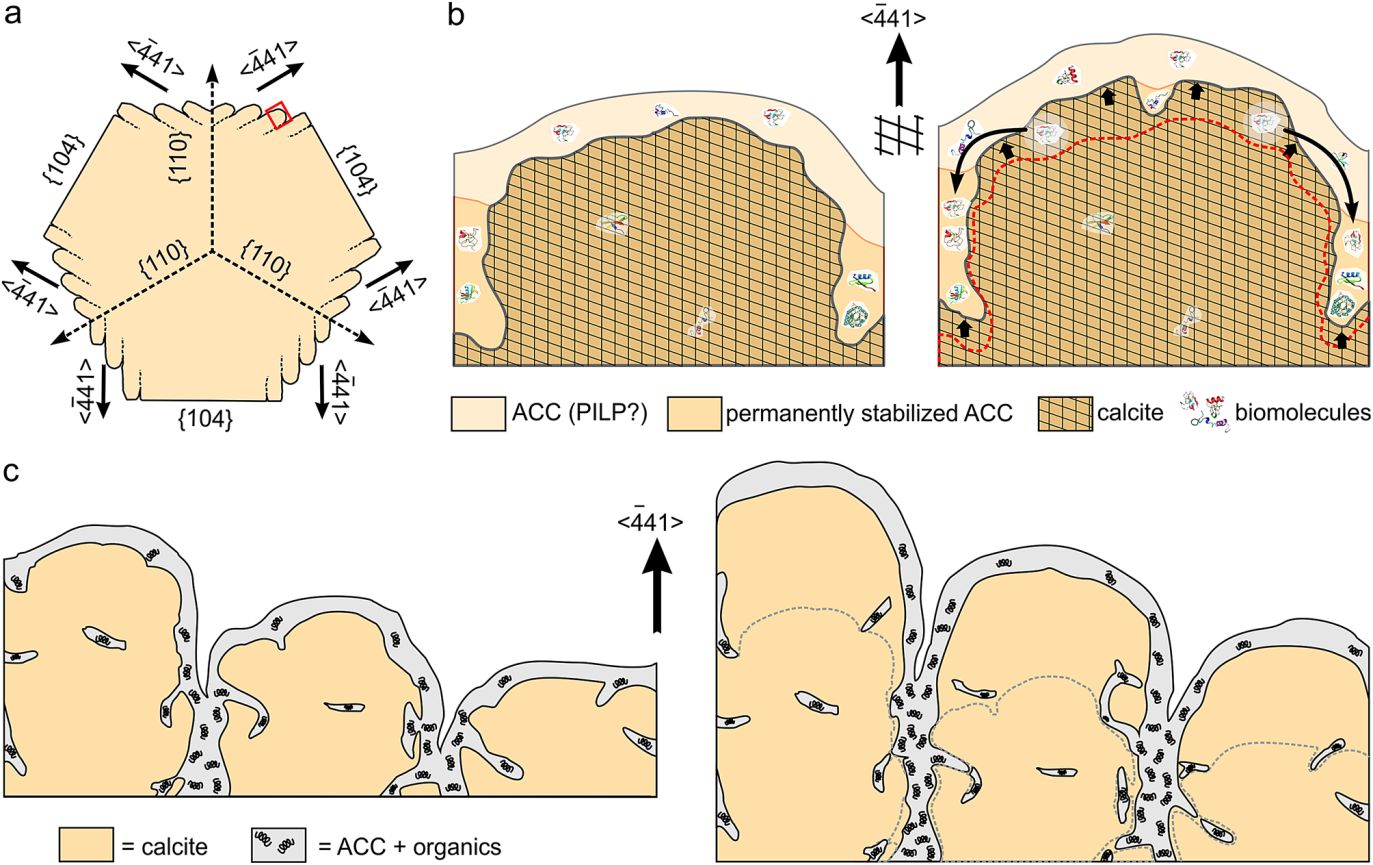
Model for the formation of elongated nanoprotrusions. (**a**) Rhombohedron forming by the elongation of nanoprotrusions, seen from the *c*-axis. (**b**) Two stages in the formation of a nanoprotrusion (area framed in (**a**)) growing along <− 441> . The crystalline phase grows at the expense of a thin ACC cortex which contains biomolecules (left). When, with growth (small thick arrows in the right panel), the crystallization front reaches the position of the biomolecules, these are expelled sideward due to the crystallization force (long arrows in the right panel). The lateral accumulations of biomolecules lead to permanently stabilised thin ACC deposits. (**c**) On a larger scale, this process produces a series of parallel nanoprotrusions elongated along <− 441> . The left and right sketches correspond to consecutive growth stages. The figure was generated with the software CorelDRAW Home & Student X8 (https://www.coreldraw.com/la/product/home-student/) by AGC.

Our model contemplates the crystallization of calcite from an amorphous precursor, which is a widely known process in biominerals, particularly in calcium carbonate-based biominerals (e.g.^21,26,27,34^). This precursor must be a mixture of ACC and organic molecules, which extends in the form of a nanometric cortex onto the growth front. This precursor may be in a liquid (polymer-induced liquid precursor, PILP^26,66,67^) phase, which is, however, not yet demonstrated in biominerals. During growth, ACC will crystallise into calcite across a crystallisation front. At the same time, new components (whether ions or nanoparticles, or both as suggested in^38^) will aggregate to the amorphous cortex, which, in this way, retains a certain thickness. During crystallization, organic molecules will become incorporated within the calcite as intracrystalline elements. The possibility of absorption of organic molecules will decrease with bond strength along the different crystallographic directions. This is particularly true for the PBCs of calcite, <010> , <42− 1> , and, especially, <− 441> , which is the strongest one^65^. Accordingly, biomacromolecules will hardly be incorporated along <− 441> and will be expelled sidewards (Fig. 8a,b), with the development of continuous crystalline nanorods in this direction (Fig. 8c). Biomacromolecule migration is facilitated if the amorphous precursor is a PILP. This is how nanoprotrusions develop and grow. The molecules expelled due to the force of crystallization will tend to accumulate at the peripheries of the nanoprotrusions and eventually stabilise tiny, pellicle-like volumes of ACC, which, upon further growth, will become occluded within the crystals (Fig. 8b,c). In this way, the biphase nanostructure extends within the crystal interior (Fig. 8c). Considering the extreme thinness of the amorphous pellicles, the external surface roughness of grains (Figs. 3a–c, 5, and Supplementary Fig. S4) mostly corresponds to the crystallization front of the biomineral.

Nanoprotrusions confer grains a characteristic cauliflower-like morphology, which is self-similar at different length scales, so that it is difficult to distinguish a particular unit size (Fig. 3b,c). These morphologies remarkably resemble those of amorphous or poorly crystalline materials, such as thin films growing from vapor at relatively low temperatures^68^. As atoms or ions arrive, they stick to the surface due to their very low mobility, forming tiny clusters. There is competition between neighbouring clusters with different heights, as the tallest ones are able to collect more atoms. This results in a very rough surface containing very small clusters (typically about 1–10 nm), corresponding to the surface diffusion length of atoms. In a similar way, the diffusion of ions or nanoparticles on the growth front of the barnacle’s biogenic calcite might be limited and thus they cluster, forming nanoprotrusions. The observed morphology of the amorphous front must be atomically rough and provides more kink sites to incorporate ions, thus speeding the growth rate^69^. Note that the proponents of the CPA hypothesis invoked ion-by-ion aggregation concomitant with particle aggregation^38^. All in all, the formation of an amorphous growth front could contribute to the surface roughness.

An important fact is the mechanism of growth of the neatly smooth {104} faces. These faces initiate at or close to the centre and spread towards the edges with the addition of nanoprotrusions at their periphery. When the nanoprotrusions reach the height of the {104} face they stop growing and their surfaces level off, thus contributing to the expansion of the face (Figs. 2e,f, and 3e,f, and 4d–k). The transition from rough to smooth surfaces could also be due to a partial dissolution–recrystallization of nanoprotrusions. After partial dissolution, increased ion surface diffusion could favour a restructuring of the surface resulting in the formation of smooth very low energy faces typical of inorganic calcite crystals (e.g.^64,70^). The observed growth mode by peripheral aggregation of nanoprotrusions differs from the classical crystal growth in which the development of rhombohedral forms is produced by spiral-growth or bidimensional nucleation occurring in low supersaturation conditions^71^. In these conditions, there is a high probability for the occurrence of screw dislocations in the centre of the faces. The generated steps move towards the edge, and the crystal face remains incomplete. Although barnacle’s incomplete rhombohedra are reminiscent of those generated by spiral growth, the former grow in a non-classical way and screw dislocations are never observed. Moreover, most grains lack flat faces.

## Summary and conclusions

The grains forming the microstructure of the wall plates of *A. psittacus* have a conspicuous nanostructure made of nano-sized protrusions, which elongate parallel to the edges of the calcite rhombohedron (<− 441> directions), which are the strongest PBCs in calcite. We hypothesise that the formation of elongated nanoprotrusions takes place during the crystallization of the precursor ACC into calcite because organic molecules are expelled from <− 441> PBCs laterally. These biomacromolecules accumulate in adjacent regions, thereby stabilizing small volumes of ACC permanently, which, with the growth of the crystals, will become occluded within the crystal, which ends up being a mixture of a crystalline and a minority amorphous phase (which we call biphase nature here). Accordingly, the surface roughness of the biogenic calcite of barnacles is a crystallographic feature. This hypothesis is consistent with biomineral growth from an amorphous precursor. A similar explanation holds true for biogenic aragonite, in which nanoprotrusions also elongate along the strongest PBC of aragonite^22,24^ (the <100> direction). It can, most likely, apply to all biominerals displaying surface roughness.

The surface roughness has traditionally been explained as resulting from the process of attachment of ACC particles to the crystal surface, which later crystallised in agreement with the crystal lattice (CPA hypothesis). The proponents of the hypothesis^38^ referred to this nanostructure as nano-particulate and defined it as “composed of spherical particles a few tens of nanometres in diameter” (their p. 5420). The presence of individual particles is not evident upon examination of the complex fractal-like growth surfaces. Our model for the formation of the surface roughness is not contrary to a CPA process, but our conclusion is that the nanoprotrusions do not correspond in size or shape with any original precursor nanoaggregate. Alternatively, the biphase nature and the surface roughness of biominerals provides evidence for crystallization from an amorphous precursor. Since direct evidence for CPA is hitherto based exclusively on experiments in in vitro systems, new evidence for this process in biological systems must be pursued.

Within the context of the CPA hypothesis, the use of the terms (nano) particle/particulate, granule/granular and alike have become a common trend because they both refer to discrete entities, which later aggregate to constitute the biocrystal. We advise the employment of merely morphological terms (protrusion or similar) to avoid this connotation.

## Methods

### Material

Specimens of the balanomorph *A. psittacus* (family Balanidae, subfamily Megabalaninae) were sampled alive in Isla Santa María, some 30 km NW of Antofagasta (ca. 23.4°S, northern Chile). Specimens of *Perforatus perforatus* (family Balanidae, subfamily Concavinae), were collected alive near Almuñécar (southeast Spain), and those of *Austrominius modestus* (Tetraclitoidea, Austrobalanidae), came from Navidad (central Chile).

### SEM

Ultrasonicated complete specimens, as well as disarticulated wall plates, were cleaned by immersion in commercial bleach (~ 5% active chlorine) for 30–60 min. Fractures and polished sections (previously etched for 2–3 min with 4% EDTA), were also bleached for 4–5 min. A few samples were heavily etched with 5% HCl for 25–30 min. All samples were carbon-coated (Emitech K975X carbon evaporator) and observed in the field emission SEMs (FESEMs) Zeiss Auriga and FEI QemScan 650 F of the Centre for Scientific Instrumentation (CIC) of the University of Granada (UGR). Some isolated plates were also analysed with the Ultra high-resolution FESEMs ThermoFisher Scientific Apreo and Verios G4, housed at NanoPort Europe (Eindhoven, The Netherlands).

### EBSD

Two wall plates of a specimen of *A. psittacus* were resin-embedded (Vosschemie GTS polyester resin) and sectioned in horizontal and vertical directions across the median axes. Sections were polished on diamond-impregnated discs with grit sizes 3, 1 and 0.75 µm in a Struers Planopol 2 polishing machine. Final polishing with colloidal silica ensued. Samples were coated with 2 nm of carbon in a BAL-TEC MED 020 electron beam evaporator to reduce charging. Analyses were carried out in a Zeiss Auriga CrossBeam Workstation (operated at 10 kV), equipped with an Oxford Instruments Nordlysnano EBSD. Areas from the sheath interior were preferentially mapped. Their locations on the samples can be seen in Supplementary Fig. S6. The step sizes were set between 0.5 and 1 μm. Post-process was done with the analysis software HKL CHANNEL5 (Oxford Instruments). The most relevant information is provided by colour maps and pole figures. All instruments are housed in the CIC (UGR).

### TEM

After freeze-drying, a lateral plate was removed and its surface scraped with a wooden tool. Fragments were ground in an agate mortar and the powder was suspended and sonicated in pure ethanol. The solution was dropped onto lacy carbon copper grids and air-dried for subsequent TEM observation. Measurements were acquired using a double Cs corrected JEOL JEM-ARM200F TEM (Max Planck Institute of Colloids and Interfaces, Potsdam) equipped with a cold-field emission gun and a Gatan OneView camera. Imaging was performed in TEM mode at 200 kV, 5 µA emission current and 500 µs exposure time using low dose technique (focusing and acquisition carried out in different sample areas). Nanodomains were indexed by Fast Fourier Transform (FFT) analysis. An example of colour reconstruction of crystalline areas based on FFT indexation is provided in Supplementary Fig. S7.

### AFM

Analyses were performed directly on the growth surfaces of primary septa and on the inner surfaces of plates. We used an AFM Park Systems NX20 (CIC, UGR) in tapping mode while displaying height, amplitude and phase signals. We used a cantilever MikroMasch NSC14 (K = 5 N/m, F = 160 kHz). Images were subsequently analysed using Park Systems XEI 4.3 software.

### XRD

A small piece of the wall plate (~ 2 cm^2^) was gently ground to fine powder with an agate mortar and pestle and mixed with silicon powder (20 wt%). Analyses were done with a Panalytical Xpert Pro X-ray powder diffractometer (CIC, UGR) (Cu Kα; 2Theta range: 4° to 120°; 0.013°step size; 200 s per step). The percentage of amorphous was determined by Rietveld refinement using TOPAS 5.0 software (Bruker, Germany), considering calcite and silicon as the internal standard (spiked 20 wt %). The analyses were done in three replicates.

### TGA and DSC

About 25 mg of powdered shell material were analysed with a TGA and DSC coupled system from METTLER-TOLEDO (model TGA/DSC1). A heating rate of 5 °C/min in air was used for registering the TGA/DSC scans. The analyses were done in three replicates.

## Supplementary information

Supplementary Figures.
