# Supplementary material for: Origin of the biphase nature and surface roughness of biogenic calcite secreted by the giant barnacle Austromegabalanus psittacus
Source: Sci Rep. 2020 Oct 8;10:16784. doi: 10.1038/s41598-020-73804-8 (PMC7544902; doi:10.1038/s41598-020-73804-8)
Supplement: Supplementary file 1 — Supplementary Figures. [file 41598_2020_73804_MOESM1_ESM.pdf]

Origin of the biphase nature and surface roughness of biogenic calcite secreted by the giant barnacle *Austromegabalanus psittacus*

Antonio G. Checa[a,b], Elena Macías-Sánchez[c,d], Alejandro B. Rodríguez-Navarro[e], Antonio Sánchez-Navas[e], Nelson A. Lagos[f]

[a]*Departamento de Estratigrafía y Paleontología, Universidad de Granada, 18071 Granada, Spain*

[b]*Instituto Andaluz de Ciencias de la Tierra, CSIC-Universidad de Granada, 18100 Armilla, Spain*

[c]*Radboud University Medical Center, Radboud Institute for Molecular Life Sciences, 6500 HB Nijmegen, The Netherlands*

[d]*Max Planck Institute of Colloids and Interfaces, 14476 Potsdam, Germany*

[e]*Departamento de Mineralogía y Petrología, Universidad de Granada, 18071 Granada, Spain*

[f]*Centro de Investigación e Innovación para el Cambio Climático, Universidad Santo Tomás, Santiago, Chile*

Supplementary Figures S1 to S7

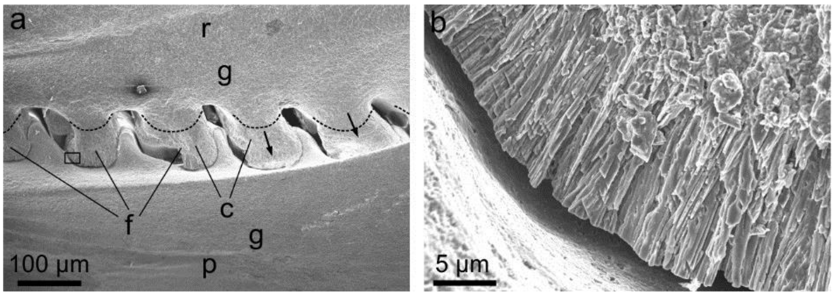

Figure S1. The fibrous microstructure of the crenulations of the growth margin of the radius. (**a**) View of the radius-paries contact. The radius growth margin develops a series of crenulations, which grow toward the paries (arrows). Their growth ends are made by fibrous material. The broken lines indicate the approximate boundary between the granular and fibrous microstructures. (**b**) Detail of the fibrous microstructure of a crenulation (small area framed in **a**). c= crenulation; f= fibrous microstructure; g= granular microstructure; p= paries; r= radius. The figure was generated with the software CorelDRAW Home & Student X8 (https://www.coreldraw.com/la/product/home-student/) by AGC.

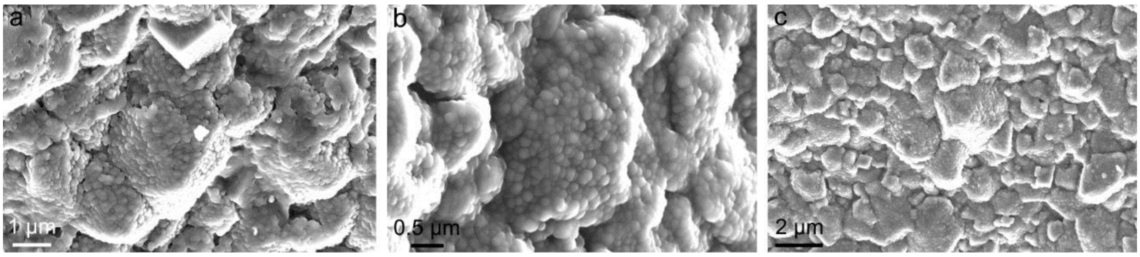

Figure S2. Surface roughness of calcitic grains of the plates of additional cirripeds. (**a**, **b**) *Perforatus perforatus*. (**c**) *Austrominius modestus*. The figure was generated with the software CorelDRAW Home & Student X8 (https://www.coreldraw.com/la/product/home-student/) by AGC.

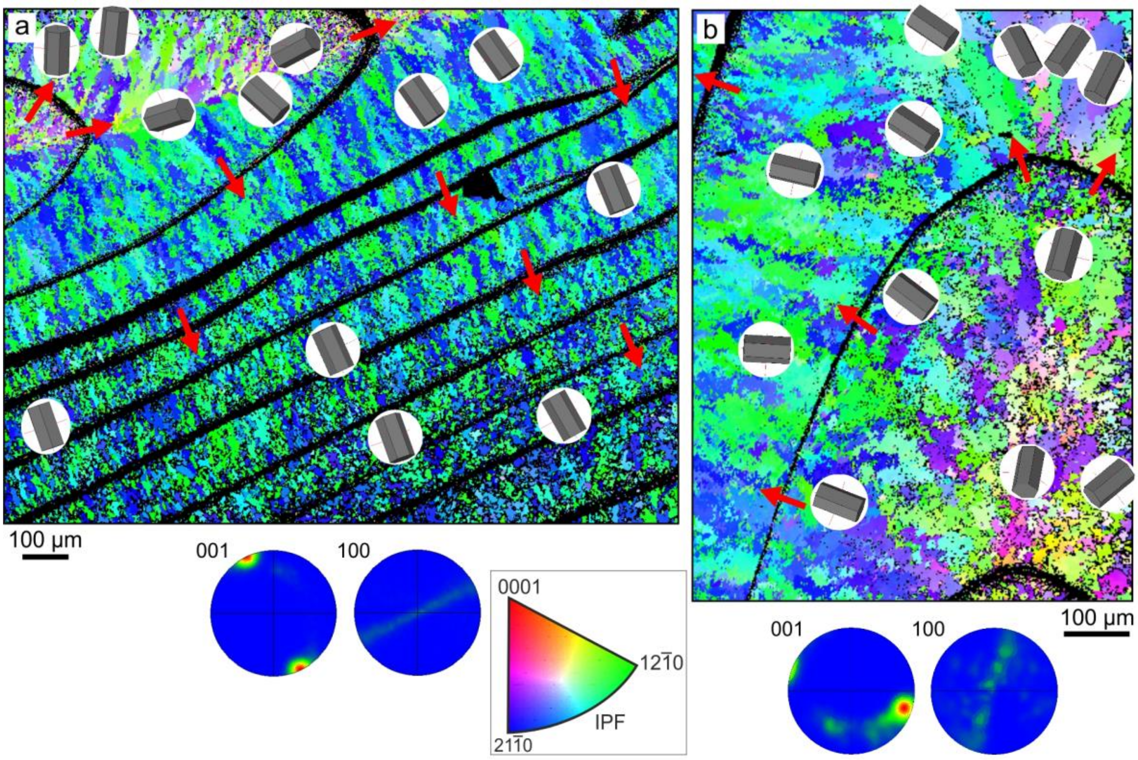

Figure S3. EBSD analysis of the granular microstructure. (**a**) Map of a horizontal section of the sheath. (**b**) Map of a vertical section of the sheath. CCRs elongate perpendicular to the growth episodes (black lines). The red arrows indicate local growth directions. The *c*-axes of unit cells are parallel to the direction of elongation of the CCRs and perpendicular to the growth episodes, i.e. parallel to the local growth directions. This trend is also indicated by the distributions of the 001 pole figure maxima. The distribution of 100 poles indicates no preferential orientation of the *a*-axes. IPF, inverse pole figure (orientation colour key) valid for the three maps. Maps, pole figures, unit cells and IPF were generated with the software AZtecHKL (Oxford Instruments) (https://nano.oxinst.com/products/aztec/aztechkl) and the composition was made with CorelDRAW Home & Student X8 (https://www.coreldraw.com/la/product/home-student/) by AGC.

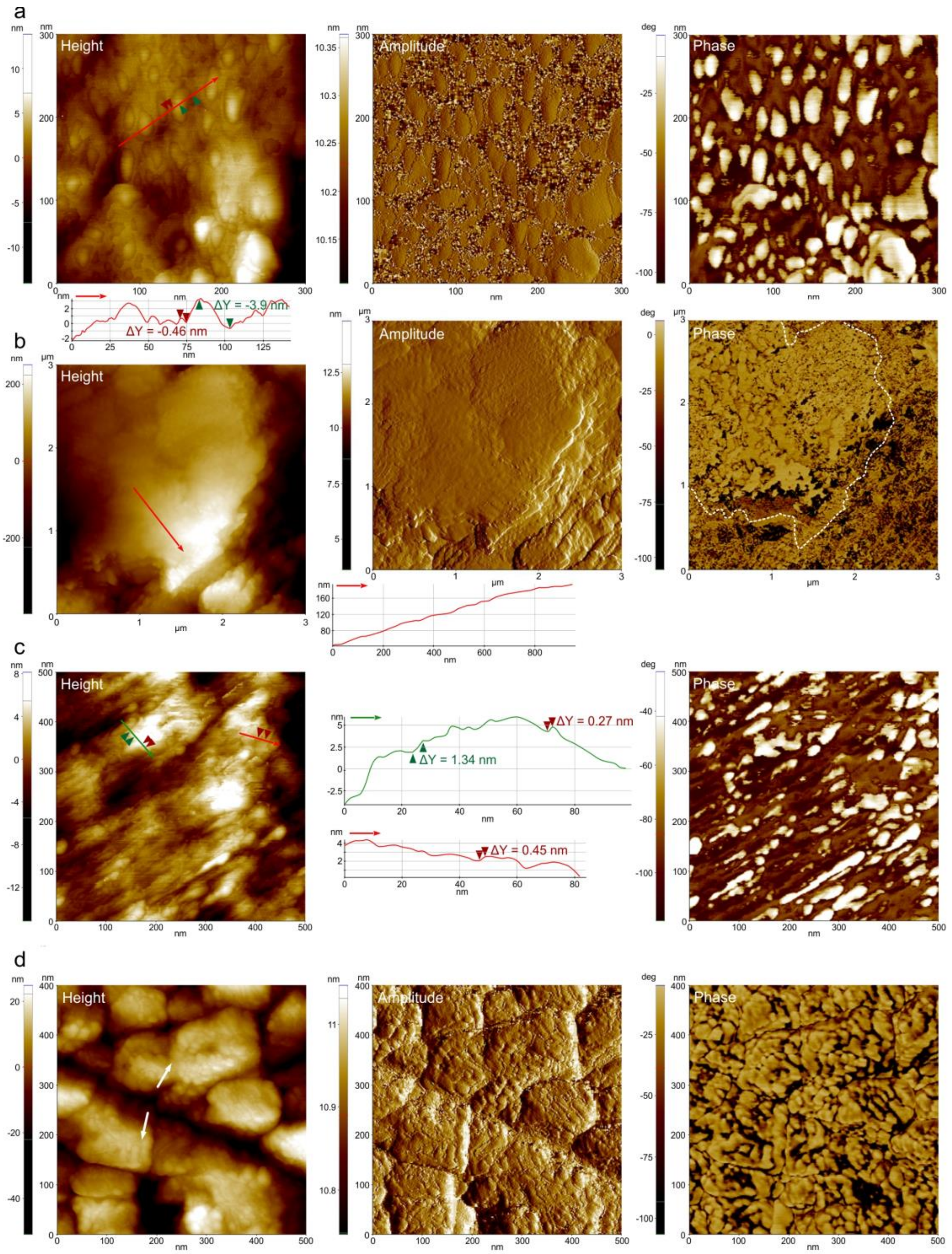

Figure S4. AFM analysis of the surfaces of granules. (**a**) Rhombohedral surface with extensive high contrast cover. The height differences indicated in the profile correspond to the thickness of a pellicle (ΔY value in red) and the height of a lump (ΔY value in green). (**b**) Rhombohedral surface (outline indicated in the phase image) with almost absent high contrast cover (compare **a** and **b** phase images). The height profile reveals the flatness of the surface. (**c**) View of a non-rhombohedral surface made of elongated lumps with extensive high-contrast coverage (phase image). The height differences (ΔY) indicated in the profiles correspond to the thicknesses of pellicles. (**d**) Detail of a line of divergence of lumps. Arrows in the height image indicate the orientation of the diverging lumps. The individual panels were generated with the software Park 4 Systems XEI 4.3 (https://park-systems-xei.software.informer.com/) and the assemblage was

made with CorelDRAW Home & Student X8 (https://www.coreldraw.com/la/product/home-student/) by AGC.

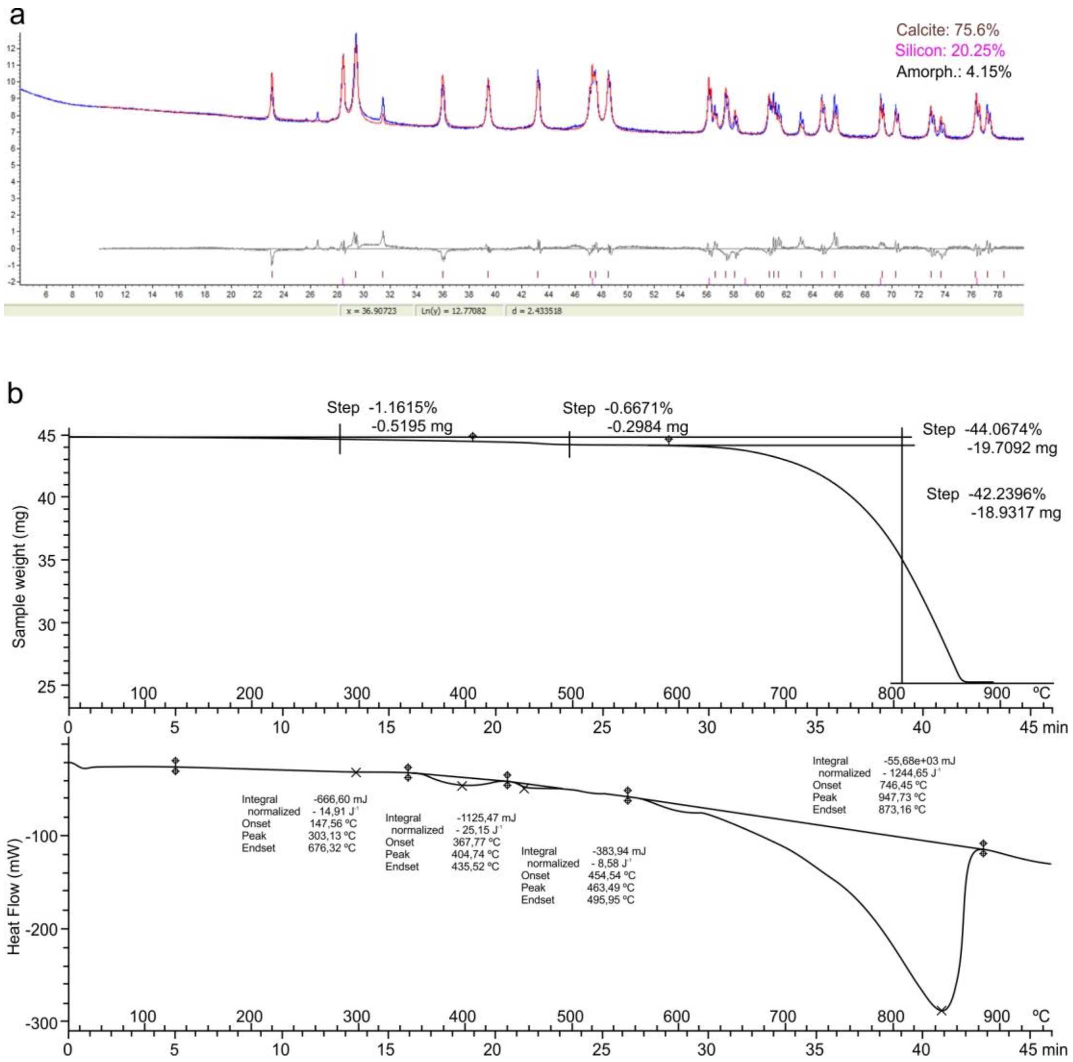

Figure S5. Determination of ACC by means of XRD and TGA-DSC. (**a**) XRD pattern of *A. psittacus* plate material spiked with 20 % silicon used as internal standard for Rietveld refinement analysis. The blue diagram is the recorded diagram (calcite+silicon), the red diagram is the calculated diagram and the lower dark grey diagram is the difference between the experimental and calculated diagrams. (**b**) TGA (upper) and DSC (lower) curves of the same sample of *A. psittacus*. The initial two weight drops (up to 500 °C) correspond to the loss of water and organic matter. The figure was generated with the software CorelDRAW Home & Student X8 (https://www.coreldraw.com/la/product/home-student/) by AGC.

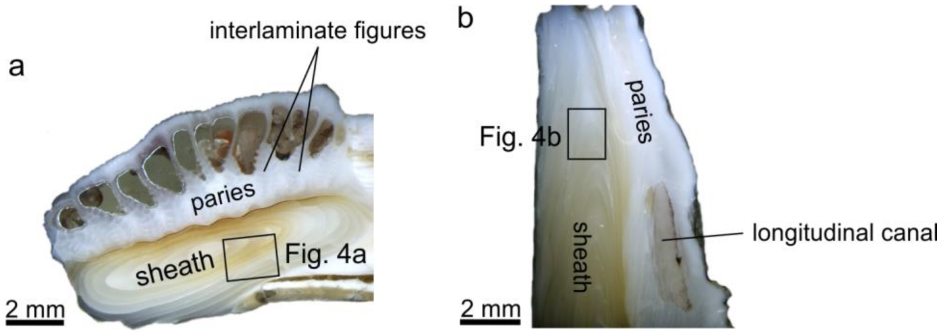

Figure S6. Position of the EBSD maps of Figure S3 on the polished samples. (**a**) Horizontally sectioned plate. (**b**) Vertically (dorsoventrally) sectioned plate. The figure was generated with the software CorelDRAW Home & Student X8 (https://www.coreldraw.com/la/product/home-student/) by AGC.

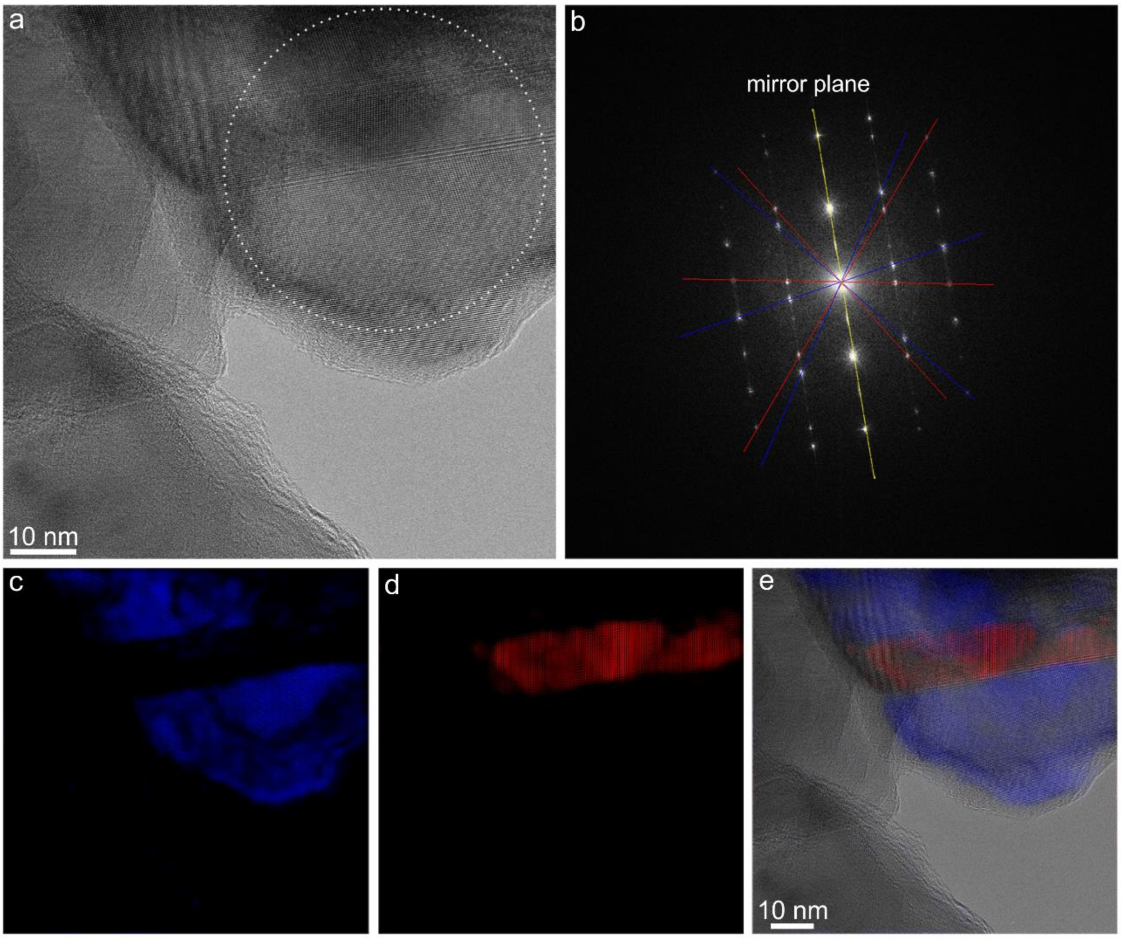

Figure S7. Example of a Fast Fourier Transform (FFT) analysis of a twin domain. (**a**) HRTEM image of a crystalline area. (**b**) FFT from the area encircled in **a**. It confirms the presence of a twin (non-indexed). Crystallographically coherent reflections are selected using a mask. (**c**) By applying an inverse FFT to the selected reflections (blue lines in **b**), the source areas of those

diffracted beams are revealed. (**d**) Reconstructions similar to that in **c** for the reflections along the red lines in **b**. (**e**) Overlap of the colour reconstructions (**c**, **d**) on the HRTEM image (**a**). The figure was generated with the software Gatan Digital Micrograph 3.4 (https://www.gatan.com/products/tem-analysis/gatan-microscopy-suite-software) by EMS, and CorelDRAW Home & Student X8 (https://www.coreldraw.com/la/product/home-student/) by AGC.
